# Disrupted Sleep and Circadian Rhythms in Schizophrenia and Their Interaction With Dopamine Signaling

**DOI:** 10.3389/fnins.2020.00636

**Published:** 2020-06-23

**Authors:** Anna Ashton, Aarti Jagannath

**Affiliations:** Sleep and Circadian Neuroscience Institute, Nuffield Department of Clinical Neurosciences, University of Oxford, Oxford, United Kingdom

**Keywords:** circadian, sleep, schizophrenia, dopamine, clock, SCRD

## Abstract

Sleep and circadian rhythm disruption (SCRD) is a common feature of schizophrenia, and is associated with symptom severity and patient quality of life. It is commonly manifested as disturbances to the sleep/wake cycle, with sleep abnormalities occurring in up to 80% of patients, making it one of the most common symptoms of this disorder. Severe circadian misalignment has also been reported, including non-24 h periods and phase advances and delays. In parallel, there are alterations to physiological circadian parameters such as body temperature and rhythmic hormone production. At the molecular level, alterations in the rhythmic expression of core clock genes indicate a dysfunctional circadian clock. Furthermore, genetic association studies have demonstrated that mutations in several clock genes are associated with a higher risk of schizophrenia. Collectively, the evidence strongly suggests that sleep and circadian disruption is not only a symptom of schizophrenia but also plays an important causal role in this disorder. The alterations in dopamine signaling that occur in schizophrenia are likely to be central to this role. Dopamine is well-documented to be involved in the regulation of the sleep/wake cycle, in which it acts to promote wakefulness, such that elevated dopamine levels can disturb sleep. There is also evidence for the influence of dopamine on the circadian clock, such as through entrainment of the master clock in the suprachiasmatic nuclei (SCN), and dopamine signaling itself is under circadian control. Therefore dopamine is closely linked with sleep and the circadian system; it appears that they have a complex, bidirectional relationship in the pathogenesis of schizophrenia, such that disturbances to one exacerbate abnormalities in the other. This review will provide an overview of the evidence for a role of SCRD in schizophrenia, and examine the interplay of this with altered dopamine signaling. We will assess the evidence to suggest common underlying mechanisms in the regulation of sleep/circadian rhythms and the pathophysiology of schizophrenia. Improvements in sleep are associated with improvements in symptoms, along with quality of life measures such as cognitive ability and employability. Therefore the circadian system holds valuable potential as a new therapeutic target for this disorder.

## Introduction

Schizophrenia is a severe psychiatric disorder, it is a leading cause of disability affecting nearly 1% of the global population (Vos et al., 2017; Moreno-Küstner et al., 2018). It is heterogeneous in nature, characterized by a combination of positive symptoms, including hallucinations and disorganized speech, negative symptoms such as social withdrawal, and cognitive symptoms. The neural mechanisms underlying it are unclear, but the most widely accepted hypothesis is aberrant dopamine (DA) signaling. Sleep and circadian rhythm disruption (SCRD) is a common feature of the disorder and is closely associated with symptom severity and patient quality of life (Cosgrave et al., 2018a). It is now considered to be more than symptomatic and is thought to contribute to the pathophysiology of schizophrenia (Manoach et al., 2016; Cosgrave et al., 2018b).

**FIGURE 1:**
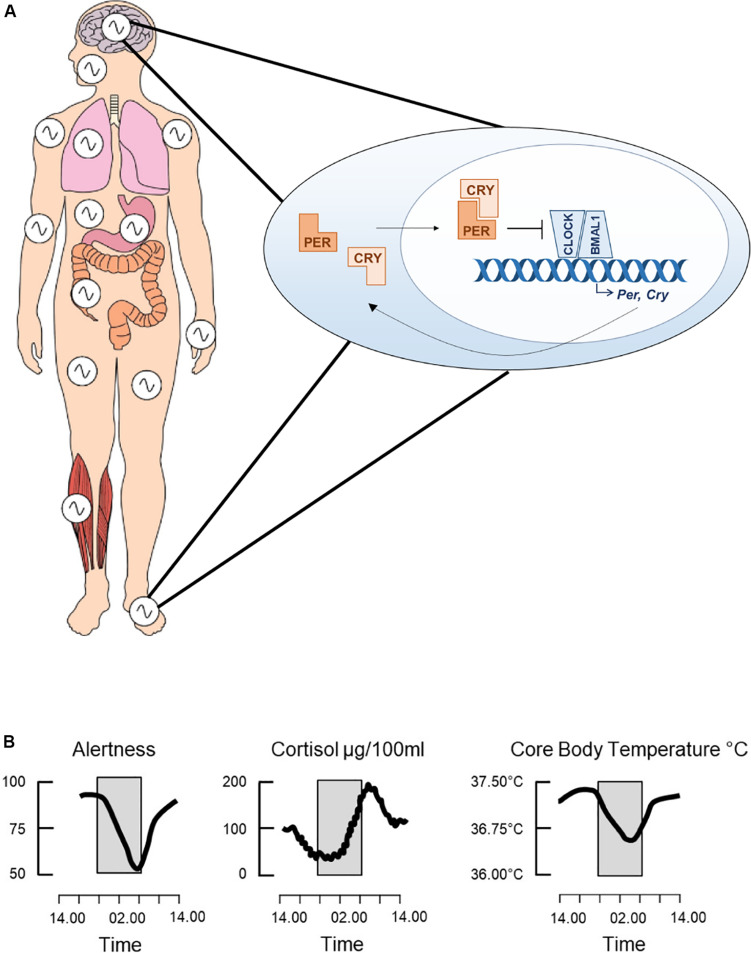
The circadian clock. The clock consists of a molecular transcriptional-translational feedback loop whereby the transcription factors, CLOCK and BMAL1, heterodimerize and induce expression of the core clock genes, *Per* and *Cry* **(A)**. PER and CRY then feedback onto CLOCK and BMAL1 by inhibiting their transcriptional activity. This feedback loop cycles with a period of around 24 h and is present in cells throughout the body, thus driving both the central circadian clock and peripheral clocks. Collectively these drive daily rhythms in physiology and behavior, such as changes in alertness levels, secretion of the stress hormone cortisol and core body temperature **(B)**. Adapted from Jagannath et al. (2013), used with permission.

Circadian rhythms are oscillations in physiology and behavior of around 24 h, having evolved across phylogeny to enable an organism to anticipate daily changes in the external environment, such as the light/dark cycle. The endogenous circadian clock drives these rhythms. In mammals, this consists of a molecular transcriptional-translational feedback loop (TTFL) in which the transcription factors CLOCK and BMAL1 induce expression of the clock genes *Per1/2* and *Cry1/2*, which then feedback to repress CLOCK and BMAL1 transcriptional activity (Figure 1A; Reppert and Weaver, 2002). This core clock machinery is present in almost every cell throughout the body (Dibner et al., 2010). Peripheral clocks are aligned by the master circadian pacemaker in the suprachiasmatic nuclei (SCN) of the hypothalamus, resulting in a coordinated network of cell autonomous circadian oscillators driving rhythmic outputs (Figure 1B). The SCN receives light input directly from the retina, which acts as the primary time cue, or “zeitgeber,” for entrainment of the clock to changes in the external environment. The sleep/wake cycle is one such rhythm under the control of the circadian clock, but it is also driven by homeostatic sleep pressure, which accumulates during wakefulness and dissipates with sleep (Borbely and Achermann, 1999). Numerous neurotransmitters are involved in the regulation of sleep, including DA, which is an established regulator of the sleep/wake cycle, exerting a potent wake-promoting activity (Eban-Rothschild et al., 2018). There is also evidence that DA has roles in the regulation of circadian rhythms, such as through modulation of SCN entrainment and behavioral oscillators.

The mechanisms underlying SCRD in schizophrenia are currently unclear. However the involvement of DA in both schizophrenia pathogenesis and the sleep and circadian systems suggests that it may be involved. Here, we examine the interplay of SCRD with DA in schizophrenia, assessing the role of DA in the regulation of sleep and circadian rhythms, as well as the circadian influence on DA itself.

## Sleep and Circadian Rhythm Disruption in Schizophrenia

Sleep and circadian rhythm disruption is widespread and well documented in schizophrenia. Indeed sleep disruption has been noted to be associated with mental health problems for over a century and the prevalence of SCRD in schizophrenia was reported to be as high as 80% (Wirz-Justice et al., 2001; Chouinard et al., 2004; Afonso et al., 2011; Bromundt et al., 2011). These showed that patients with schizophrenia had disrupted sleep/wake cycles as measured by wrist actigraphy and melatonin and cortisol profiles, which are markers of endogenous rhythmicity in patients with schizophrenia. However, as most of these studies lacked suitable control subjects, it was difficult to draw conclusions about confounding effects from medication and socio-economic factors including employment status. Wulff et al. (2012), were amongst the first to systematically evaluate circadian disruption in a cohort with schizophrenia against healthy unemployed controls and showed all of the 20 patients assessed had significant SCRD, but none of the controls. Importantly, the patients showed wide heterogeneity in phenotypes, including advanced/delayed phase, non-24 h rhythms that were not entrained by the light/dark cycle and fragmented and irregular sleep patterns, perhaps reflecting the heterogeneity in the disease itself. The nature and extent of SCRD in schizophrenia have been covered in previous reviews and we point the reader to these for further information (Wulff et al., 2010; Jagannath et al., 2013; Chan et al., 2017; Cosgrave et al., 2018b).

### Causal Role of Sleep and Circadian Rhythm Disruption

Recent studies provide evidence that sleep disruption may indeed play a causal role in the development of psychosis. It is clear that SCRD can precede the appearance of psychosis on the one hand, and exacerbate negative outcomes on the other. Fragmented sleep and blunted circadian rhythmicity of behavior have been observed in adolescents and young adults at risk for psychosis (Castro et al., 2015) and SCRD predicted the severity of psychosis symptoms and psychosocial impairment at 1 year follow-up in adolescents with clinically high risk for psychosis (Lunsford-Avery et al., 2017). With similar findings in bipolar disorder (Ng et al., 2015), now often considered to be on the same spectrum as schizophrenia, further long-term studies are required to assess the predictive power of SCRD in early diagnosis. Even in healthy subjects, psychotic episodes can be predicted by poor perceived sleep quality in combination with reduced sleep duration (Cosgrave et al., 2018b). Further, sleep deprivation, or restricted sleep to mimic insomnia in healthy subjects leads to symptoms associated with schizophrenia such as reduced prepulse inhibition, increased delusions and hallucinations, and psychotic episodes (Petrovsky et al., 2014; Reeve et al., 2018).

Disrupted sleep in schizophrenia is associated with poor quality of life, higher rates of relapse and suicide (Hofstetter et al., 2005; Pompili et al., 2009; Palmese et al., 2011), and the stabilization of sleep can have beneficial effects. A controlled trial administering cognitive behavioral therapy (CBT) to reduce insomnia in a cohort of over 3000 university students had the concomitant outcome of reducing the appearance of paranoid delusions and hallucinations in those subjects whose insomnia was reduced (Freeman et al., 2017). This approach has also been used in patients with schizophrenia where improvements in sleep are associated with reduced persecutory delusions and hallucinations (Myers et al., 2011; Waite et al., 2016). Pharmacotherapy for sleep stabilization includes traditional sedative-hypnotics such as benzodiazepines, and second generation sedating antipsychotics such as quetiapine, olanzapine and risperidone improve sleep quality, and pharmacotherapy for sleep also correlates with an improvement in negative symptoms (Kajimura et al., 1995; Yamashita et al., 2004; Kluge et al., 2014). Pharmacotherapy is administered with caution however, as many sedative-hypnotics impair slow-wave sleep and REM sleep patterns, sleep parameters which may already be impaired in schizophrenia.

Clinical studies examining melatonin use as an add-on therapy in schizophrenia suggest that it may also be effective at ameliorating sleep disruption in this disorder (reviewed in Bastos et al., 2019). Melatonin is a key hormonal output of the central clock, its rhythmic release acts to signal circadian time to the brain and peripheral tissues. Administered alongside antipsychotic medication, it results in improved sleep measures including sleep efficiency and duration (Shamir et al., 2000; Kumar et al., 2007). Furthermore, animal studies have demonstrated that it reduces schizophrenia-like behaviors in mice, suggesting that it may also be effective at treating symptoms such as cognitive impairment and social withdrawal (da Silva Araújo et al., 2017; Onaolapo et al., 2017). However, studies which have looked at the effect of melatonin on the psychotic symptoms in patients, although limited, show conflicting results. A reduction in symptom severity has been reported with add-on treatment of both melatonin (Modabbernia et al., 2014) and the melatonin receptor agonist, ramelteon (Mishra et al., 2020). In contrast, Romo-Nava et al. (2014) did not detect any improvement resulting from melatonin administration over placebo, although this study also included patients with bipolar disorder. Inconsistencies in melatonin efficacy may be due to deficiencies in its target regions or its receptors. Indeed, a polymorphism in the MT1 melatonin receptor gene is associated with schizophrenia (Park et al., 2011), indicating that the receptor expression may be altered in some patients.

Together the evidence from these studies suggests SCRD could indeed play a causal role in the development of schizophrenia and that the stabilization of SCRD has beneficial outcomes. As an extension, these findings suggest the SCRD could be a manifestation of a dysfunctional circadian system, which could underlie aspects of the pathophysiology of schizophrenia.

Genome-wide association studies have associated mutations in circadian clock genes with an increased risk of schizophrenia; single nucleotide polymorphisms (SNPs) in *CLOCK*, *PER2*, *PER3*, *RORB*, and *TIMELESS* have been associated with the disorder as assessed within relatively small groups of patients, numbering a few hundred (Takao et al., 2007; Mansour et al., 2009; Zhang et al., 2011). Whilst these associations with clock genes have not been replicated in larger studies and meta-analyses (Schizophrenia Working Group of the Psychiatric Genomics Consortium, 2014; Pardiñas et al., 2018; Rees et al., 2019; Wang et al., 2019), these larger studies have implicated SNPs in genes with a role in dopaminergic/glutamatergic neurotransmission, mitochondrial function and immunity. All of these pathways also directly feed into the regulation of circadian rhythms or are regulated by the clock; highlighting multiple points of convergence with circadian rhythms in the pathophysiology of schizophrenia. For example, dopaminergic regulation of the clock is described in detail below and there is a growing body of literature on elevated neuroinflammation in the prefrontal cortex in schizophrenia. This has now been linked to increased nuclear factor-κB (NF-κB) transcriptional activation (Volk et al., 2019), and NF-κB function is regulated by CLOCK, with *Clock* deficient mice showing significantly reduced NF-κB activation in response to immunostimuli (Spengler et al., 2012).

Circadian disruption could result from aberrant synaptic networks and connectivity within the brain, which characterize both schizophrenia and bipolar disorder (Chai et al., 2011), or from a deficit at the level of clock gene expression. There is evidence for both. Blind-drunk (*Bdr*) is a mouse model of synaptosomal-associated protein (Snap)-25 exocytotic disruption that displays schizophrenic endophenotypes. Their circadian rhythms are fragmented and phase advanced, and there is some evidence that this arises from disruption of synaptic connectivity within the SCN, thus disrupting rhythmic outputs (Oliver et al., 2012). On the other hand, skin fibroblasts isolated from patients with chronic schizophrenia lose rhythms in the expression of the clock genes *CRY1* and *PER2* when compared with healthy controls (Johansson et al., 2016), and *PER1/2/3* and *NPAS2* mRNA levels were reported to be altered in white blood cells from schizophrenia patients (Sun et al., 2016). These studies show a deficit at the level of the molecular clock in schizophrenia that is cell autonomous, much like the clock itself. This has been replicated recently by a study analyzing gene expression in the human dorsolateral prefrontal cortex in schizophrenia and control subjects (Seney et al., 2019). By factoring time-of-death into transcriptome analysis, Seney et al. (2019) showed diurnal rhythms in gene expression of very different sets of genes in two groups, with circadian signaling showing strong rhythmic patterns in the control participants but not the schizophrenia patients. In contrast, mitochondrial signaling genes were rhythmic in the patients, but not the controls. The implications of these findings are as yet unclear, but point toward a mechanistic role for circadian rhythm, and consequently circadian disruption in schizophrenia.

## Mechanisms Underlying Sleep and Circadian Disruption in Schizophrenia: Role of Dopamine?

The mechanisms underlying SCRD in schizophrenia are currently unclear. However, there is evidence from numerous studies that DA plays a role in the regulation of both sleep and the circadian system. Given that aberrant DA signaling is the longest standing hypothesis for schizophrenia pathogenesis, this may underlie the SCRD in the disorder.

### Dopamine in Wake and Sleep

Dopamine is one of the major regulators of sleep and wakefulness. Evidence from numerous studies spanning mammalian species and non-mammalian models, demonstrates the importance of DA in sleep control across phylogeny (Eban-Rothschild et al., 2018). The dopaminergic neurons in the ventral tegmental area (VTA) and dorsal raphe nucleus (DRN) are key components of the neuronal circuitry for the regulation of sleep and wake states in mammals, owing to their potent wake-promoting activity (Lu et al., 2006; Eban-Rothschild et al., 2016, 2018; Cho et al., 2017; Oishi et al., 2017; Yang et al., 2018). Selective optogenetic stimulation of VTA DA neurons in mice can induce behavioral arousal responses during anesthesia (Taylor et al., 2016), and induce wakefulness during NREM sleep, even following sleep deprivation when homeostatic sleep pressure would be high (Eban-Rothschild et al., 2016). Similarly, activation of DA neurons in the DRN induces wakefulness during NREM and REM sleep, while their chemogenetic inhibition reduces wakefulness and increases NREM sleep duration (Cho et al., 2017).

Dopamine neurons in both these regions, the VTA and DRN, exhibit differential firing patterns between sleep/wake states, although there are differences in the activity patterns between the two regions. In the DRN, DA populations are primarily wake-active, and interestingly the net increase in their activity at wake onset positively correlates with the duration of the subsequent wake episode (Cho et al., 2017), as it does in the striatum (Dong et al., 2019). Therefore DA levels at wake onset may determine the length of the following wake period, and subsequently influence sleep/wake cycles. While in the VTA, there is also higher DA activity during wake, with an increase in population burst firing compared to NREM sleep, however the activity of these neurons is further increased during REM sleep compared to wake (Dahan et al., 2007; Eban-Rothschild et al., 2016), suggesting distinct roles of these two DA regions in arousal. The increase in firing of VTA neurons is accompanied by an increase in extracellular DA levels in their projection target regions, the nucleus accumbens and prefrontal cortex (Léna et al., 2005), leading to sleep/wake-state dependent changes in DA levels.

However, not all DA populations are arousal-inducing, there is also evidence for a sleep-promoting role. Selective lesions of DA neurons in the substantia nigra, which project to the dorsal striatum, leads to increased wakefulness, reduced NREM and REM sleep, and fragmentation of sleep/wake states in rats, while optogenetic stimulation of these neuron terminals leads to increased NREM sleep (Qiu et al., 2016). Furthermore, low doses of D_2_ receptor (D_2_R) agonists have been reported to induce sedative effects and increase sleep in humans and rodents (Monti et al., 1989; Hamidovic et al., 2008; Laloux et al., 2008). Although this may be due to activation of pre-synaptic autoreceptors and the subsequent inhibition of DA neurons in arousal-promoting regions (Monti and Monti, 2007).

Further evidence for the role of DA in arousal is provided by the effects of stimulants (Boutrel and Koob, 2004). Genetic and pharmacological studies have demonstrated that the primary mechanism underlying the wake-inducing action of the stimulant modafinil is an increase in DA (Wisor et al., 2001; Qu et al., 2008). This is the preferred treatment for hyper-somnias such as narcolepsy, and is thought to act through inhibition of DA reuptake. Similarly, selective DA reuptake inhibitors have also been shown to promote wakefulness in rodents and dogs (Nishino et al., 1998; Luca et al., 2018).

Collectively these studies have demonstrated that DA signaling has a key role in the regulation of sleep/wake states. Generally it serves a wake-promoting role, however its effects are bidirectional and can promote both wakefulness or sleep depending on the brain region and receptor subtypes involved. Its role in sleep/wake control is therefore complex, and the precise molecular and neuroanatomical mechanisms through which it acts are still unclear, yet it is evident that normal DA signaling is important for stable sleep/wake cycles.

### Dopamine and the Circadian Clock

The sleep/wake cycle is also under circadian control to ensure that its timing is aligned with the external light/dark cycle, and the two systems are closely linked. There is growing evidence that DA is also an important modulator of the circadian system.

Dopamine signaling is involved in the functioning of two key components of the circadian system: the retina and the SCN. In the retina, it is necessary for functions including light adaptation and visual acuity (Jackson et al., 2012). Importantly, it is also one of the main modulators of retinal circadian activity (Green and Besharse, 2004) and can influence the core circadian clock, controlling the amplitude of rhythmic clock gene expression (Yujnovsky et al., 2006). There is also evidence that DA via D_2_R drives rhythmic expression of the photopigment melanopsin in intrinsically photosensitive retinal ganglion cells (ipRGCs; Sakamoto et al., 2005), which are the primary photoreceptors responsible for light input to the SCN (Peirson and Foster, 2006). Therefore retinal DA is a key modulator of non-visual light detection, and may be important for photic entrainment of the master clock. Indeed, Doi et al. (2006) demonstrated that genetic deletion of D_2_R in mice leads to a deficient masking response to light, whereby light suppresses locomotor activity during the dark period when the mice are usually active. However, the D_2_R null mice did exhibit normal entrainment to a light/dark cycle, and photic responses in the SCN and pineal gland were intact. These data suggest that D_2_R is required for behavioral responses to light, but not for other non-visual photic responses such as photic entrainment of the SCN. Although in this study D_2_R was deleted throughout the brain, so while retinal DA signaling may be involved, the involvement of DA signaling in other regions cannot be ruled out.

In the SCN itself, there is a high number of dopaminergic neurons, with *Drd1*-expressing neurons comprising approximately 60% of cells in rodents (Smyllie et al., 2016). Early studies demonstrated that DA is required for entrainment of the developing SCN, acting to synchronize fetal-maternal circadian rhythms (Mendoza and Challet, 2014). More recently, studies have suggested that DA modulation of the central clock persists into adulthood (Jones et al., 2015; Landgraf et al., 2016; Smyllie et al., 2016; Grippo et al., 2017). Studies report that *Drd1*-expressing SCN neurons are able to entrain and set the period of circadian rhythms in mice (Jones et al., 2015; Smyllie et al., 2016). However, this does not provide direct evidence for a role of DA itself, as the *Drd1*-positive cells overlap with the majority of arginine vasopressin (AVP) and vasoactive intestinal peptide (VIP) neurons, other key SCN subpopulations involved in entrainment.

However, Grippo et al. (2017) did demonstrate DA modulation of the adult SCN clock via D_1_R signaling. They showed that DA signaling in the mouse SCN is required for normal re-entrainment following a jet lag-like phase shift in the light/dark cycle (Grippo et al., 2017). Retrograde tracing suggested that this was mediated by direct DA innervation from the VTA, with enhancement of this DA input leading to accelerated re-entrainment. Interestingly, this facilitation of resynchronization by DA required light input, in line with previous studies that showed that administration of D_1_R agonist alone during constant conditions does not elicit any phase shifts in hamster (Duffield et al., 1998; Grosse and Davis, 1999). This suggests that direct midbrain DA innervation of the SCN can modulate the light-responsiveness of the master clock, enabling quicker re-entrainment. Importantly, this study also demonstrates that the level of DA tone can have a direct impact on the central clock, and therefore provides a mechanism whereby aberrant DA signaling, such as an inappropriately timed elevation in DA tone, could influence the master clock. However, this study did not detect changes in several circadian parameters following modulation of SCN DA signaling, such as the period length during constant conditions and the phase angle of entrainment. Therefore, it appears that while DA via D_1_R has a modulatory role of the light-responsiveness of the clock, it is not necessary for its normal functioning. Studies looking at additional measures of the circadian clock other than locomotor activity are required to elucidate the involvement of DA in the clock, given the essential role of DA in motor function (Ryczko and Dubuc, 2017).

There is also evidence for a role of DA in clock regulation outside of the SCN. As previously discussed here, DA is a major modulator of the retinal clock, in addition to this it also acts on the striatal clock. The dorsal striatum is heavily innervated by dopaminergic projections from the substantia nigra. Evidence from *in vivo* pharmacological and lesioning studies suggest that DA signaling via D_2_R is required for an intact rhythm in *Per2* expression in the dorsal striatum (Hood et al., 2010). Extracellular DA levels were found to oscillate here over 24 h with a peak during night, preceding the peak in *Per2* expression. However, this was measured in rats maintained under a light/dark cycle, so it is unclear whether this is an endogenous circadian rhythm that persists under constant darkness or whether it requires light input.

There is also evidence that DA can regulate circadian rhythms in hormone production and behavior. It is thought to be necessary for the rhythmic release of prolactin in the arcuate nucleus of the hypothalamus (Bertram et al., 2010), and exerts an inhibitory modulation of melatonin synthesis in the pineal gland (González et al., 2012). Melatonin is an important output of the SCN for the regulation of circadian and seasonal rhythms throughout the body, and also feeds back to the SCN itself to modulate the central clock. DA is also thought to control rhythms in activity; lesioning of dopaminergic regions leads to a lengthening in the period of wheel-running and drinking activity during constant conditions (Isobe and Nishino, 2001; Tanaka et al., 2012). However, as DA itself is under circadian control (Chung et al., 2014) as discussed below, it may be that in these cases DA is an intermediate signal between the circadian clock and clock-controlled processes, rather driving the circadian rhythms itself. Nevertheless, it appears to be an important component in the regulation of circadian-controlled processes.

However, DA is in fact able to act independently of the master clock to regulate rhythms in behavior through DA-driven pacemakers which are entrainable by two non-photic stimuli: food and methamphetamine. The DA-enhancing psychostimulant, methamphetamine, can induce circadian rhythmicity in locomotor activity in the absence of an SCN, via the methamphetamine-sensitive circadian oscillator (MASCO; Tataroglu et al., 2006). This finding led to the identification of a ‘dopamine ultradian oscillator’ (DUO), whereby ultradian oscillations in DA drive an ultradian rhythm in locomotor activity, which persists in *Bmal1* null mice and is therefore independent of the circadian clock (Blum et al., 2014). Interestingly, this study used *Slc6a3-/-* mice to model a hyper-dopaminergic phenotype, due to their deletion of DA transporter (DAT) for DA re-uptake, a potential model for schizophrenia. Elevated DA in these mice led to aberrant ultradian activity rhythms with fragmented daily activity, which mirrors the disruptions to the rest/activity cycle observed in patients with schizophrenia (Wulff et al., 2012), suggesting a causal role for DA in these symptoms. This highlights the central role that DA has in the regulation of activity rhythms, and importantly, demonstrates a potential mechanism through which aberrant DA can lead to abnormalities in rest/activity rhythms seen in schizophrenia.

Dopamine is also necessary for the activity of the food entrainable oscillator (FEO). In the absence of an SCN this drives food anticipatory activity (FAA), whereby an increase in locomotor activity precedes mealtimes by 1 to 3 h (Liu et al., 2012; Gallardo et al., 2014). DA signaling via D_1_R in the dorsal striatum mediates this rhythmic activity, suggesting that DA is required for daily rhythms in feeding activity. While the underlying mechanisms are still being elucidated, these SCN-independent oscillators likely feed into the SCN to regulate rhythmic behavior in concert with the central circadian clock.

### Interaction of Dopamine With Other Neurotransmitter Systems

**FIGURE 2:**
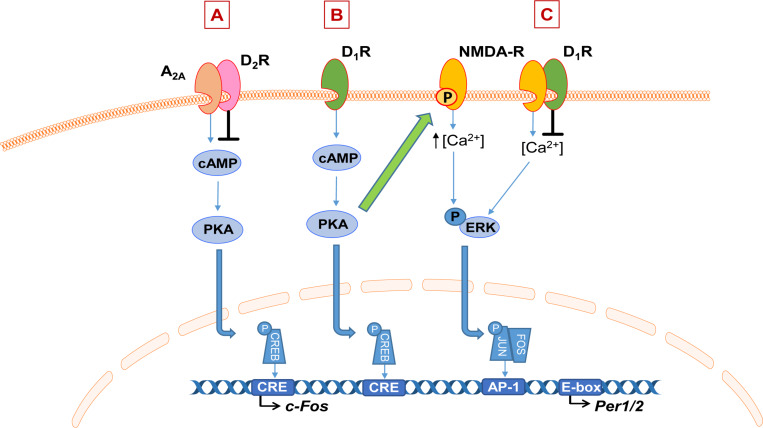
Schematic of dopamine interaction with adenosine and NMDA signaling. **(A)** Dopamine (DA) D2 receptors (D_2_R) heteromerize with adenosine 2A receptors (A_2__A_R) to inhibit their downstream signaling via the cAMP-PKA pathway, impacting gene expression such as immediate early genes. **(B)** Example of DA-mediated enhancement of NMDA receptor (NMDA-R) signaling. Activation of the dopamine D1 receptor (D_1_R) activates the cAMP-PKA pathway which leads to phosphorylation of the NR2B subunit of the NMDA receptor, which is thought to enhance NMDA-dependent calcium influx and therefore downstream responses such as ERK phosphorylation and subsequent transcriptional activation. On the other hand, heteromerization of D_1_R or D_2_R with NMDA receptors inhibits NMDA-mediated currents **(C)**.

There is evidence that DA interacts directly with other neurotransmitter systems that are involved in the regulation of sleep and the circadian clock, namely adenosine and glutamate via NMDA receptors. There is an antagonistic interaction between DA and the G-protein-coupled adenosine receptors via both D_1_R and D_2_R. The D_1_R heteromerize with the A_1_R, resulting in an inhibitory action of adenosine to dampen DA receptor response (Ciruela et al., 2011). Whereas D_2_R form a complex with the A_2__A_ subtype of adenosine receptors, resulting in a reciprocal antagonistic interaction whereby D_2_R activation can also dampen the A_2__A_R response (Figure 2A). Activation of D_2_R is thought to inhibit A_2__A_R activation of the cAMP-PKA pathway, therefore leading to inhibition of adenosine-induced protein phosphorylation and gene expression, such as of immediate-early genes (IEGs; Ferré et al., 1997; Svenningsson et al., 2000). Adenosine is involved in sleep/wake control; the accumulation of extracellular adenosine during wake and its dissipation in sleep is thought to underlie homeostatic sleep drive, or “sleep pressure” (Landolt, 2008). Furthermore, the stimulant caffeine acts primarily through antagonism of A_2__A_R (Huang et al., 2005). Therefore, DA-mediated modulation of adenosine signaling through receptor heteromerization is a potential mechanism through which DA can modulate sleep and wakefulness, although the role of this interaction in sleep control is yet to be elucidated.

It is well-established that DA modulates NMDA receptor signaling; the two receptors can form functional heteromers and their downstream signaling pathways also interact at multiple levels (Perreault et al., 2014; Klein et al., 2019). Their interaction is complex; DA can have both a potentiating and inhibitory effect on the NMDA receptor response. Activation of D_1_R can enhance NMDA-mediated responses (Cepeda et al., 1993, 1998; Levine et al., 1996). Evidence suggests this is through D_1_R-dependent phosphorylation of the NR2B subunit of the NMDA receptor, which is thought to enhance NMDA-dependent calcium influx and therefore downstream responses such as ERK signaling (Figure 2B; Pascoli et al., 2011; Murphy et al., 2014). On the other hand, heteromerization of both the D_1_R and D_2_R with NMDA receptors leads to inhibition of NMDA currents (Figure 2C; Perreault et al., 2014). NMDA receptor activation is one of the primary mediators of light input into the SCN and therefore a key mechanism for photic entrainment of clock gene expression in the master clock (Golombek and Rosenstein, 2010). Together, these represent intriguing pathways for future investigation through which aberrant DA signaling may impact neurotransmitter systems that are central to the regulation of the sleep and circadian system.

## Effects of Antipsychotic Medication on Sleep and Circadian Rhythms

Given the influence of DA on sleep and the circadian clock, it would be expected that antipsychotic drugs which act to antagonize DA receptors will also influence these systems. Generally both typical and atypical antipsychotics can improve sleep, although their effects on sleep architecture in patients are not yet fully understood. Studies using both subjective measures and polysomnographic assessments have reported an increase in total sleep time and sleep efficiency, in parallel with clinical improvements (reviewed in detail in Cohrs, 2008). However there are variable effects of different medications on specific sleep parameters, such as the percentage of REM sleep. This is likely due to their different pharmacological profiles; they each act on multiple neurotransmitter systems in addition to dopamine, including serotonin, noradrenaline, and histamine (Miyamoto et al., 2005). Rodent studies have shown that antipsychotic drugs with different receptor selectivity have differential effects on sleep/wake states (Ongini et al., 1993; Gould et al., 2016). Atypical antipsychotics generally have higher affinity for serotonin receptors, which may underlie their overall higher efficacy at improving sleep over typical antipsychotics (Cohrs, 2008). Studies on the effects of antipsychotic medication in healthy subjects have also demonstrated sleep-promoting effects, indicating that this activity is independent of schizophrenia pathology. However, in a study of subjective sleep quality in hospitalized patients, sleep disturbances were found to persist in the majority of patients undergoing antipsychotic treatment (Waters et al., 2012), demonstrating limited efficacy of antipsychotics at improving sleep in all cases. Although most of the patients in this study were on additional medication such as antidepressants or anticonvulsants, leading to potential confounds.

In addition to influencing sleep, there is also some evidence that antipsychotics affect the circadian system, with differential effects of the typical and atypical classes. The atypical antipsychotic clozapine appears to improve abnormalities in rest/activity rhythms in schizophrenia patients, in comparison to typical antipsychotics such as haloperidol (Wirz-Justice et al., 1997, 2001). These studies lacked pre-treatment baseline data however, therefore it is unclear whether haloperidol itself had a role in driving the arrhythmic rest/activity patterns in these cases. Indeed, there is evidence from studies in healthy mice to suggest that haloperidol can influence the circadian clock. Acute administration of haloperidol can induce *Per1* expression in the mouse SCN via NMDA receptors and CREB signaling (Viyoch et al., 2005). Whereas chronic treatment can suppress *Per1* expression across various brain regions (Coogan et al., 2011), but has no effect on *Bmal1* in most of these regions, suggesting that alterations in the negative arm of the core clock TTFL did not impact on the positive arm here. On the other hand, clozapine was not found to influence clock genes in a small sample study analyzing their expression in white blood cells from patients following an 8 week treatment course (Sun et al., 2016). There were no improvements in the abnormal rhythmic clock gene expression, despite the improvements in circadian rhythms in response to clozapine treatment previously reported. Overall, the actions of antipsychotic medication on the circadian system are unclear from these limited studies. Nevertheless, these findings do demonstrate the importance of appropriate timing of drug administration to correspond with the endogenous clock and sleep/wake cycle, in order to ensure that patients’ are not sleeping at the wrong time of day and to prevent further sleep and circadian disturbance.

## Influence of the Circadian Clock and Sleep on Dopamine Signaling

Studies detailed above demonstrate that DA is able to modulate the entrainment of the central circadian clock and its outputs, as well drive behavioral oscillators and modulate circadian clocks outside of the SCN. Interestingly the link between DA and the circadian clock is bidirectional, with DA also being under the control of the clock.

Several elements of DA signaling have a diurnal rhythm, including expression of DA receptors and tyrosine hydroxylase (TH), the rate-limiting enzyme in DA synthesis (Castañeda et al., 2004; Weber et al., 2004; Ferris et al., 2014; Ozburn et al., 2015; Sidor et al., 2015). Numerous rodent studies have reported diurnal changes in extracellular DA levels in various brain regions such as the prefrontal cortex and nucleus accumbens, with generally higher levels at night when rodents are active (Smith et al., 1992; Hood et al., 2010; Menon et al., 2019). Similarly, circulating DA levels in men peak during the day (Sowers and Vlachakis, 1984).

Evidence suggests that these diurnal rhythms are driven by direct regulation of DA signaling components by the circadian clock. The synthetic enzyme TH is under direct transcriptional control by both CLOCK (Sidor et al., 2015) and REV-ERBα (Chung et al., 2014); their genetic ablation leads to elevated DA signaling, with an increase in active TH and firing of dopaminergic VTA neurons (McClung et al., 2005; Chung et al., 2014). Furthermore NPAS2 directly regulates expression of the DA receptor gene *Drd3*, and is required for its rhythmic expression in the nucleus accumbens (Ozburn et al., 2015). In addition, the enzyme monoamine oxidase A (MAOA), which is required for DA metabolism, is also under circadian control; *Per2* is required for its diurnal rhythm in expression, and *Per2* mutant mice exhibit reduced MAOA activity and increased DA levels in the striatum (Hampp et al., 2008). Collectively, these studies demonstrate that DA signaling itself is rhythmic and is under direct circadian control.

There are also numerous lines of evidence that the DA system is modulated by melatonin. The MT1 melatonin receptors are expressed in dopaminergic regions of the rodent and human brain, including the VTA and nucleus accumbens where they exhibit diurnal changes in expression (Uz et al., 2005; Lacoste et al., 2015). Generally, studies have demonstrated an inhibitory effect of melatonin on the dopaminergic system, with an inhibition of DA release widely reported across species and brain regions (Zisapel and Laudon, 1982; Zisapel et al., 1982, 1985; Dubocovich, 1983; Boatright et al., 1994; Exposito et al., 1995), as well as an inhibition of dopaminergic neuronal firing (Domínguez-López et al., 2014). However at the molecular level, studies appear to indicate an enhancement of DA levels, with melatonin reported to increase TH activity (Alexiuk et al., 1996; McMillan et al., 2007), and decrease DA degradative enzyme activity (Esquifino et al., 1994; Stefanovic et al., 2016), as well as increase expression of vesicular monoamine transporter 2 (VMAT2; Stefanovic et al., 2016), which mediates synaptic release of DA. The mechanisms underlying the inhibitory action of melatonin are therefore unclear and its effects are likely to be dependent on both region and circadian time. Zisapel et al. (1985) demonstrated that melatonin inhibits dopamine in a time-dependent manner, suggesting that there is a circadian rhythm in its sensitivity to melatonin. Nevertheless, melatonin clearly influences DA signaling and therefore abnormalities in the melatonin rhythm, such as has been observed in schizophrenia, may disturb the DA system.

There are also changes in extracellular DA levels across the spontaneous sleep/wake cycle. The fluctuations vary between brain regions, but generally levels are higher during wakefulness (Léna et al., 2005; Dong et al., 2019; Menon et al., 2019). In the mouse striatum, DA levels also exhibit dynamic changes within periods of wakefulness and at sleep/wake state transitions (Dong et al., 2019). Given the potent wake-promoting activity of DA, it is currently unclear whether this correlation of DA levels with sleep/wake state is dependent on the sleep/wake cycle or whether elevated DA precedes wakefulness onset. However, there is evidence that sleep deprivation leads to an increase in DA in the nucleus accumbens (Murillo-Rodríguez et al., 2016), suggesting that sleep can modulate DA levels.

## Bidirectional Relationship Between Dopamine and Sleep and Circadian Systems

Overall, it is clear that DA and the sleep and circadian systems interact at multiple levels, and this reciprocal relationship may be a key element of schizophrenia pathophysiology. Numerous studies outlined here have demonstrated the involvement of DA in the regulation of both sleep and circadian rhythms. It is therefore implicated that alterations to DA signaling, such that occur in schizophrenia, would lead to disruptions to sleep and circadian rhythms. Indeed, elevation of DA signaling through genetic and pharmacological approaches leads to disrupted sleep and fragmented rest/activity rhythms (Wisor et al., 2001; Monti and Monti, 2007; Blum et al., 2014). Furthermore, circadian rhythm disturbances and disrupted sleep/wake cycles are common in Parkinson’s disease, in which degeneration of nigrostriatal dopaminergic neurons leads to altered DA signaling (Videnovic et al., 2014; Grippo and Güler, 2019). Therefore dysregulation of the DA system can lead to disruption to sleep and circadian rhythms, and so this may be a key mechanism underlying the SCRD in schizophrenia.

On the other hand, DA is also under the control of the circadian clock and exhibits sleep-dependent changes, therefore it is likely that SCRD will lead to dysregulation of the DA system. SCRD is a key feature of schizophrenia pathophysiology, it largely occurs independent of medication status or disease stage (Karatsoreos, 2014) and there is compelling evidence for a causal role of SCRD in schizophrenia. Indeed, studies outlined above have demonstrated that an intact clock is required for normal levels of DA activity and rhythmic expression of DA signaling components, with alterations to clock gene expression causing elevations in dopaminergic tone. Furthermore, destabilization of the sleep/wake cycle can cause aberrant DA signaling, with sleep deprivation resulting in increased DA activity (Volkow et al., 2008; Zant et al., 2011; Murillo-Rodríguez et al., 2016).

**FIGURE 3:**
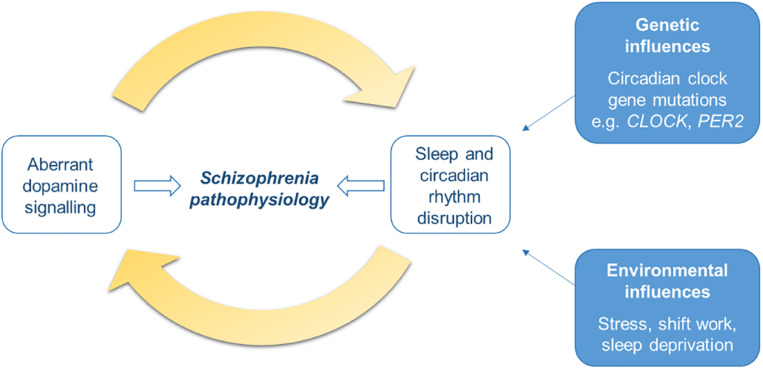
Schematic of the bidirectional relationship of dopamine signaling with sleep and circadian rhythm disruption (SCRD). The dopamine (DA) system is involved in sleep-wake control and circadian rhythms, therefore aberrant DA signaling will result in disruption to sleep and circadian rhythms. On the other hand, DA is also under the control of the circadian clock and its activity varies with sleep stage, therefore SCRD, arising from dysregulation of the DA system itself, or genetic or environmental factors, will aberrantly impact DA signaling. Dysregulation to each of these systems will exacerbate each other; disturbances to one will worsen abnormalities in the other, forming a positive feedback cycle. This process may be an important mechanism underlying schizophrenia pathogenesis and progression of the illness.

Therefore, overall the DA system and the sleep and circadian systems are key modulators of each other. The two have a bidirectional relationship whereby dysregulation of one will lead to disturbances to the other, this will likely lead to a positive feedback loop in which the two exacerbate each other (Figure 3). Given that both aberrant DA signaling and SCRD are implicated in schizophrenia pathogenesis, this interplay may be an important process in the development of the disorder. Studies assessing the outcome of early intervention to address sleep and circadian disturbances in the prodromal stages will help to elucidate the causal role of this bidirectional relationship in schizophrenia.

## Conclusion

Sleep and circadian rhythm disruption is a key feature of schizophrenia, with deficits present from the level of the molecular clock up to behavioral rhythms. Studies discussed here demonstrate that DA signaling is an important modulator of sleep and circadian rhythms, and therefore constitutes a common mechanism underlying both sleep and circadian rhythm regulation, and schizophrenia pathophysiology. On the other hand, DA is also influenced by the circadian clock and sleep, and the close reciprocal relationship of the two systems appears to be important in the development of schizophrenia. Whilst further understanding of the mechanisms underlying this complex interaction is needed, there are already several potential pathways where dysregulation of DA can lead to disturbances to sleep and circadian rhythms, suggesting that aberrant DA may lead to SCRD in schizophrenia. Nevertheless, targeting sleep and the circadian system holds valuable therapeutic potential to improve symptoms and quality of life for patients.

## Author Contributions

AA and AJ have both contributed to the writing of the manuscript. All authors contributed to the article and approved the submitted version.

## Conflict of Interest

AJ is in receipt of funding from Circadian Therapeutics Ltd. The remaining author declares that the research was conducted in the absence of any commercial or financial relationships that could be construed as a potential conflict of interest.
